# Seroprevalence of Measles Antibodies in the Population of the Olomouc Region, Czech Republic—Comparison of the Results of Four Laboratories

**DOI:** 10.3390/vaccines10020185

**Published:** 2022-01-25

**Authors:** Michal Krupka, Tereza Matusu, Helena Sutova, Katerina Wezdenkova, Renata Vecerova, Yvona Smesna, Milan Kolar, Hana Bilkova Frankova, Jana Krivankova, Miroslav Jorenek, Zdenek Novak, Milan Raska, Ondrej Holy

**Affiliations:** 1 Department of Immunology, Faculty of Medicine and Dentistry, Palacky University Olomouc, 775 15 Olomouc, Czech Republic; krupka.olomouc@gmail.com (M.K.); milan.raska@upol.cz (M.R.); 2 Mikrochem Laboratories a.s., 779 00 Olomouc, Czech Republic; tereza.matusu@michem.cz (T.M.); helena.sutova@michem.cz (H.S.); katerina.wezdenkova@michem.cz (K.W.); 3 Department of Microbiology, University Hospital Olomouc and Faculty of Medicine and Dentistry, Palacky University Olomouc, 775 15 Olomouc, Czech Republic; renata.vecerova@fnol.cz (R.V.); Yvona.Loveckova@fnol.cz (Y.S.); milan.kolar@upol.cz (M.K.); 4 AGEL Laboratories a.s., 703 84 Ostrava–Vítkovice, Czech Republic; hana.frankova@lab.agel.cz; 5 Šumperk Hospital, 787 01 Šumperk, Czech Republic; jana.krivankova@nemocnicesumperk.cz (J.K.); miroslav.jorenek@nemocnicesumperk.cz (M.J.); 6 Department of Surgery, University of Alabama at Birmingham, Birmingham, AL 35294, USA; ZDNovak@peds.uab.edu; 7 Science and Research Centre, Faculty of Health Sciences, Palacky University Olomouc, Hněvotínská 3, 775 15 Olomouc, Czech Republic

**Keywords:** measles, ELISA, LIAISON, Olomouc region, Czech Republic

## Abstract

Objectives: Although the incidence of measles has decreased globally since the introduction of regular vaccination, its frequency has increased again in recent years. The study is focused on data from the Olomouc Region in the Czech Republic analyzed in four laboratories. The obtained results were compared with already published data. Methods: The data were provided by individual laboratories in an anonymized form—age at the time of the examination, sex, and result of test. Samples were collected between June 2018 and September 2019 and evaluated on the scale positive–borderline–negative. Results: A total of 7962 sera samples were evaluated using three different methods—two types of ELISA tests and CLIA. Positive result was issued in a total of 62.6 percent of samples, but the results of individual laboratories varied widely from 55.5 to 70.8 percent. However, the same trend with the highest levels of antibodies in people born before beginning of vaccination was observed. Conclusions: Data show significantly different results depending on the individual laboratories and the detection kits used. The underestimation of the proportion of positive results can cause problems in selecting individuals for revaccination with a live vaccine, which may fail in weakly positive individuals.

## 1. Introduction

Measles is a highly contagious, acute febrile illness caused by the virus belonging to the family *Paramyxoviridae*, the genus *Morbillivirus*. Based on transmissibility, this virus is probably the most infectious human pathogen on the earth with basic reproduction number (R_0_) mostly estimated to 12–18 [1,2,3].

The virus is transmitted from person-to-person mostly by large respiratory droplets, but it can be also transmitted by smaller aerosolized particles remaining in the air for more than an hour after the infectious person has left [4].

After an incubation period of 7 to 18 days (average 10), prodromal symptoms appear as fever and at least one of the three “C”s: coryza, cough, or conjunctivitis. Subsequently, a typical morbilliform rash appears on the head, which then spreads to the body and limbs. The disease can be complicated by otitis media, diarrhea, pneumonia, encephalitis, or ocular disorders. A rare but mostly fatal delayed complication of measles is subacute sclerosing panencephalitis. It is a progressive neurological disorder manifesting several years after the measles, especially in young children, and usually ends in death within three years of the onset of the first symptoms [5,6,7].

Primary prevention by vaccination is the only effective form of protection against this disease. The measles virus strain, from which the vaccine strains were later derived, was isolated and propagated in tissue culture in 1954 [8]. The virus strain was named Edmonston, after the child from whom it was isolated. Further passage in primary renal and amnion cells and subsequently in chick embryo fibroblasts resulted in attenuated strains Moraten (syn. Edmonston–Enders) and Schwarz, which are currently the basis of European and American attenuated measles vaccines. The first attenuated vaccine based on Edmonston strain was licensed in 1963. At the same time, inactivated vaccines were being developed but provided only short-term protection [9,10,11]. Although a total of 24 genotypes of measles virus are described, they form a single serotype. Vaccination or infection with a single genotype thus provides general protection [11].

**Figure 1:**
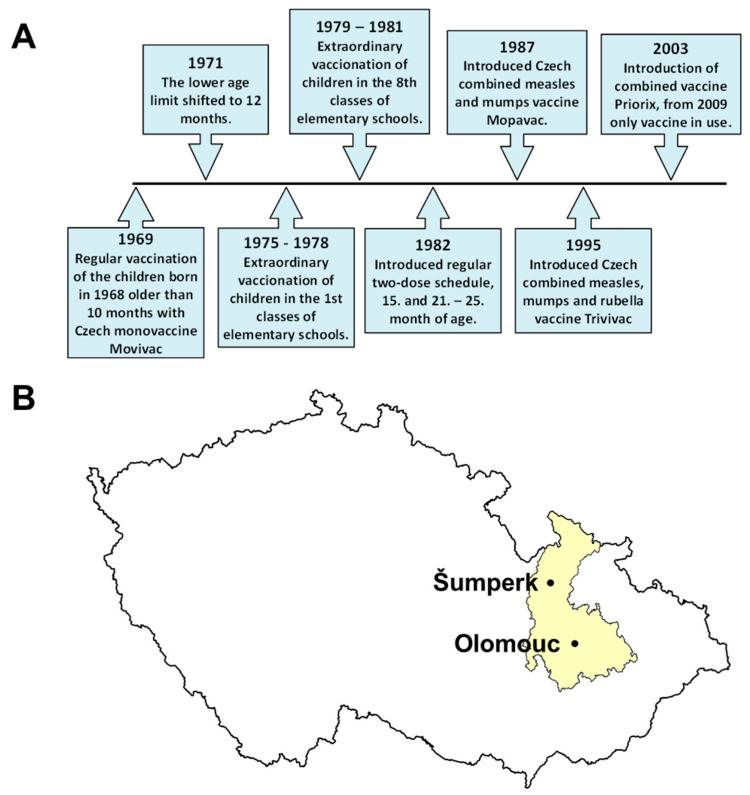
(**A**) Timeline of measles vaccination development in the Czech Republic. (**B**) Map of the Czech Republic with the marked Olomouc region (yellow) and the cities where the laboratories that provided the results for the study are located.

**Figure 2:**
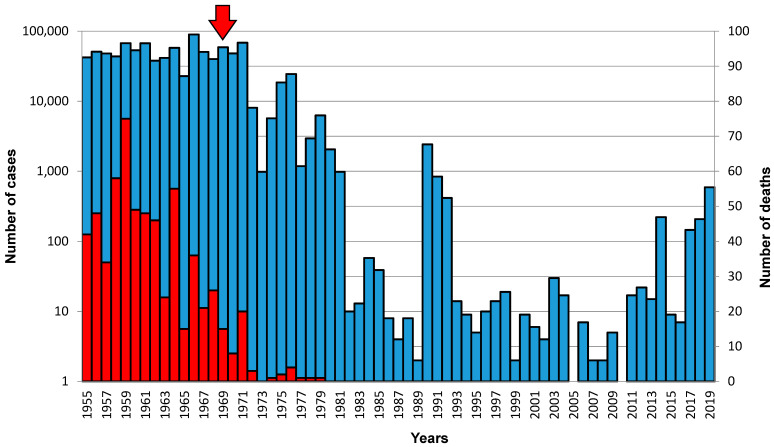
Cases of measles (blue, please note the logarithmic scale) and deaths caused by them (red) in the Czech Republic from 1955 to 2019 (Source: The National Institute of Public Health, Prague and [13]). Arrow indicates the beginning of the compulsory vaccination.

In the former Czechoslovakia (divided in 1993 to Czech and Slovak republics), regular measles vaccination was started in 1969 using a local manufacturer’s vaccine Movivac based on the strain Schwarz. The development of the vaccination schedule is shown in Figure 1. Currently, in the Czech Republic, the measles component is administered as a part of the mandatory combined measles–mumps–rubella vaccine. The first dose is given at 13–18 months, the second at 5–6 years of age [12]. The introduction of vaccination has led to a rapid decline in the incidence. The number of reported infections in Czech Republic fell from 58,973 in 1969 to 2053 in 1980 [13] and later dropped to almost zero (Figure 2). No measles deaths have been reported also since 1980. The number of reported infections began to increase again after 2010. In 2014, an epidemic broke out in the Ústí nad Labem Region following the import of an infection by a traveler from India. The infection was detected in 186 people, including 88 healthcare professionals [14]. In 2017, an epidemic appeared in the Moravian–Silesian Region, where 130 people fell ill. Both epidemics were caused by the B3 genotype strains, formerly typical for the African and Middle Eastern regions [15]. In 2019, the number of reported cases reached 590, the WHO revoked the status of “measles-free country”, and the Czech Republic lost measles elimination status (together with Albania, Greece, and the United Kingdom). The upward trend in measles occurrence is also a problem in many other European countries. According to the WHO, 82,596 cases were reported in the European region in 2018, compared with 7884 in 2009. In this year, 47 out of 53 countries in European region were affected, and 72 children and adults died due to measles infection. The epidemic is spreading mainly in Ukraine (more than 50,000 cases), Romania, Italy, France, and Greece. Worldwide, more than 140,000 people died of measles in 2018, predominantly in the Indian subcontinent and Africa. This is a significant decrease compared to 535,000 deaths in 2000 but a slight increase compared to 139,000 in 2010.

There are several possible causes of the recurrence of the frequency of infection discussed, including secondary vaccine failure caused by waning of humoral immunity in adults, decreased vaccination coverage, and increasing mobility of persons associated with high accessibility of air transport. However, it is probably a combination of all these factors in different proportions. The greatest risk for the future is considered to be declining vaccination rates due to loss of public confidence in immunization in developed countries or destabilization of health care due to violent conflicts in some African and Asian countries. The situation may also be adversely affected by limited access to healthcare including vaccination in some regions due to the current epidemic of SARS-CoV-2.

For specific immune defense against measles, both antibodies and specific cellular mechanisms are important. It has been observed that individuals with defects in cellular immunity are at risk of a severe life-threatening infection including measles, giant-cell pneumonia, or inclusion-body encephalitis [16,17]. However, the determination of specific cellular immunity against measles is not routinely performed, and thus the examination of specific antibodies is the only used parameter for evaluation of measles immunity. Biological assays such as plaque reduction neutralization test, which show only antibodies with neutralizing activity, have the greatest informative value from the assays used to detect measles antibodies [18]. Due to their laboriousness and difficult automation, these tests have been replaced in most routine laboratories by enzyme or chemiluminescent immunoassays. However, these tests may be affected by the variable properties of commercial kits from different manufacturers, including different definitions of positive, cut-off, and negative values.

In the presented work, we took advantage of the increased interest in testing of measles antibodies levels caused by the worsening of the epidemiological situation, and we collected data from four major laboratories in the Olomouc Region. The structure of the data, in addition to providing a serological overview in the region, also allowed us to make a basic comparison of different methods for determining measles antibodies—automated chemiluminescence assay and enzyme immunoassays with quantitative or semi-quantitative evaluation.

## 2. Materials and Methods

### Collection and Evaluation of Samples

The study is focused on data from the Olomouc Region, which is one of the fourteen The Nomenclature of Territorial Units for Statistics (NUTS3) units in the Czech Republic (CZ071) and includes an area of 527,154 km^2^ with 632,015 inhabitants (Figure 1B).

The data of the examined persons were provided in an anonymized form, containing only the age at the time of the examination, sex, and the test result. To prevent possible identification, the processed data did not contain dates of birth or national personal numbers. The project was approved by the Ethical Committee of the University Hospital Olomouc and by the responsible authorities of the participating organizations.

The results of tests were mainly obtained during preventative actions based on the recommendation of the Czech Ministry of Health. As clinical outcome, both negative and borderline results were issued as non-immune state, but for the purposes of the study, borderline and negative results were processed separately. All persons with antibody levels assessed as “insufficient” (negative and borderline) were offered a revaccination by one dose of combined vaccine Priorix. Samples were collected and evaluated between June 2018 and September 2019. 

The results were obtained by the following medical facilities:

AGEL a.s. is the largest healthcare provider in the Czech Republic operating 12 hospitals and a network of clinics, pharmacies, and laboratories. The AGEL laboratories provided test results from 1852 sampled participants. Samples were taken in AGEL facilities and external healthcare providers (general practitioners, outpatient specialists, vaccination centers). 

The levels of measle antibodies were evaluated by enzyme immunoassay (EIA) using detection kit Anti-Measles Virus ELISA IgG (Euroimmun, Germany). The results were presented in International Units per liter (mIU/mL) and evaluated according to the manufacturer’s manual as follows: <200 mIU/mL—negative; 200–275 mIU/mL—borderline; ≥275 mIU/mL—positive.

University Hospital Olomouc is the largest medical facility in the Olomouc Region. It is part of a network of nine teaching hospitals directly managed by the Ministry of Health of the Czech Republic. The data provided were obtained during a project aimed to assess levels of anti-measles antibodies in hospital employees [19]. Therefore, all tested persons are staff members of this organization, mostly physicians, nurses, and laboratory workers. 

Antibody levels were measured at the Department of Microbiology using the same test and cut-off values as in the AGEL laboratories; a total of 3093 samples were tested. 

Mikrochem a.s. is private laboratory company founded in 1994 by the privatization of Microbiology section of Regional Public Health Office in Olomouc. At present, the company has branches in three other towns and focuses on the laboratory diagnostics in the fields of microbiology and immunology. The laboratory provided data obtained from 2267 samples. Overall, 573 samples were taken from members of the fire department, 668 from members of the police force, and 710 from healthcare workers (mainly employees of the Olomouc Military Hospital).

Antibodies levels were measured by chemiluminescence immunoassay (CLIA) with paramagnetic microparticle solid phase using LIAISON^®^ analyzer and Measles IgG kit (DiaSorin, Italy). Results were calculated as arbitrary units per milliliter (AU/mL); the cut-off value distinguishing between the presence and absence of protective measles antibodies specified by manufacturer is 15 AU/mL. According to the WHO standard for measles serum NIBSC: 97/648, this value corresponds to 175 mIU/mL. To comply with the test instructions, the results were evaluated as follows: <13.5 AU/mL—negative result, 13.5–16.5 AU/mL—unclear (borderline) result, and ≥16.5 AU/mL—positive result. 

Šumperk Hospital is a secondary care facility with a catchment area in the northern part of the Olomouc region, comprising about 200,000 inhabitants (almost one-third of the region). The data provided were obtained by testing the hospital staff. 

Antibody levels were measured at the Microbiology laboratory of the Šumperk Hospital using a Measles IgG ELISA kit (Immunolab, Kassel, Germany). Results were evaluated in the form of the positivity index, which expresses the ratio between the absorbance of the sample and the absorbance of the standard (cut-off calibrator). Values <0.8 were evaluated as negative, 0.8–1.2 as borderline, and >1.2 as positive. The manufacturer does not state a link to the WHO standard in the test manual. Overall, 750 samples were processed in this laboratory.

## 3. Statistical Analysis

Data are presented as absolute numbers and percent prevalence (%). Continuous variables are presented as average ± standard deviation or as a median and inter-quartile range, where appropriate. Chi-square testing (or Fisher’s exact test where applicable) was used to compare frequencies and independent samples. A Student’s *t*-test or analysis of variance (ANOVA) with Tukey post hoc test was used to compare continuous variables. Mann–Whitney U-test or Kruskal–Wallis test was used in instances where a non-parametric test was required. Logistic regression modeling was used to analyze odds-ratios and association of age group and test result. All models were built separately for each laboratory method. A *p*-value of less that 0.05 was considered statistically significant in two-tail tests. All data were analyzed using SPSS v.25 package (IBM Inc., New York, NY, USA).

## 4. Results

A total number of 7962 sera samples were obtained, which is so far the largest set of data focused on measles antibodies published from the Czech Republic. Data were obtained from four laboratories in the Olomouc region using three different methods—EIA with quantitative evaluation (AGEL laboratories and University Hospital in Olomouc), EIA with evaluation using the relative index (Šumperk Hospital), and CLIA with quantitative evaluation (Mikrochem laboratories). The basic characteristics of the study population are given in Table 1. 

**Figure 3:**
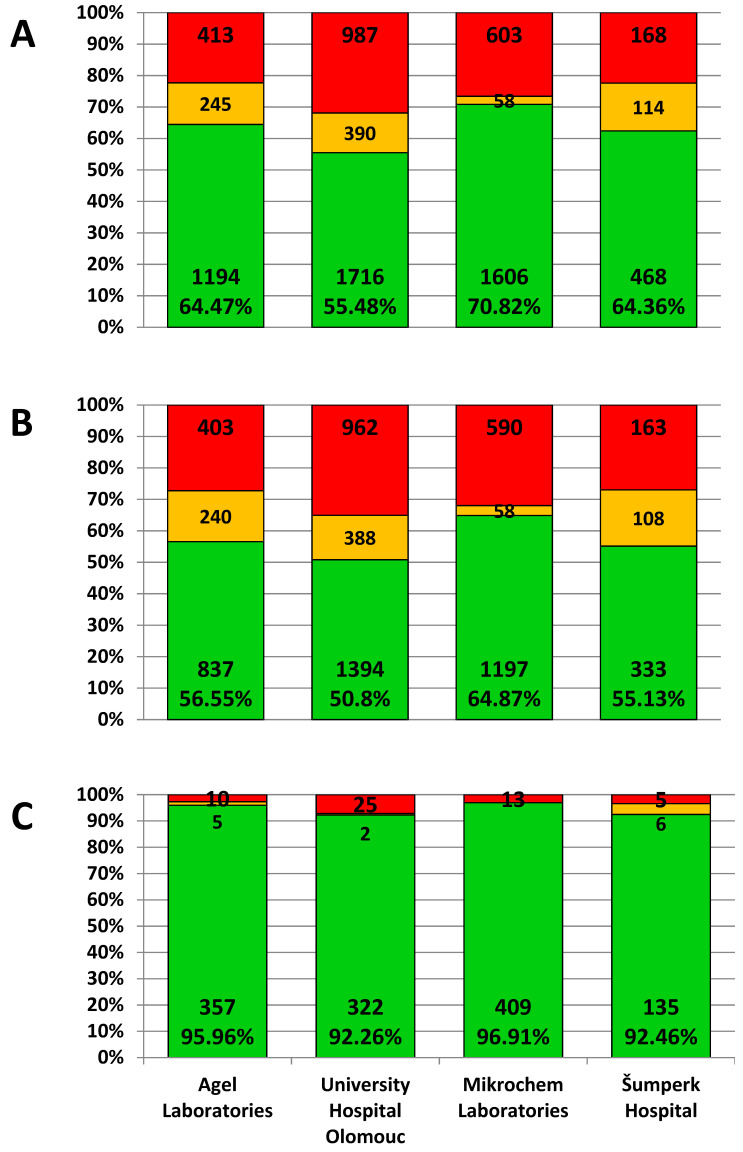
The proportion of positive (green), borderline (orange), and negative (red) results in individual laboratories. (**A**) Total values, (**B**) persons aged 52 years and younger, (**C**) persons aged 53 years and older. Using chi-square statistics performed on the entire study cohort and sub-cohorts of subjects ≤52 or ≥53 years of age (at the time of sampling), we observed significant differences in frequencies of negative, positive, and borderline values among all four labs.

When evaluated according to the instructions of the manufacturer of specific detection kits, a positive result was issued in a total of 62.59 percent of samples. For clinical purposes, borderline results were released by the laboratories as insufficient levels, as well as negative results. For individual laboratories, the proportion of positive results was as follows: AGEL laboratories—64.47%, University Hospital Olomouc—55.48%, Mikrochem laboratories—70.82%, and Šumperk Hospital 64.36% (Figure 3). Differences between all laboratories were determined to be statistically significant using the Chi-Square test (*p* < 0.001). Noteworthy is the fact that the difference between the results of two laboratories using the same detection kit is greater than the difference between the results of two laboratories using different EIA methods (AGEL vs. University Hospital—64.47 × 55.48%; AGEL vs. Šumperk Hospital—64.47 × 64.36%). There is also an obvious difference in the proportion of results evaluated as equivocal, which were less frequent when using CLIA method compared to the other groups of results obtained by the EIA methods.

**Figure 4:**
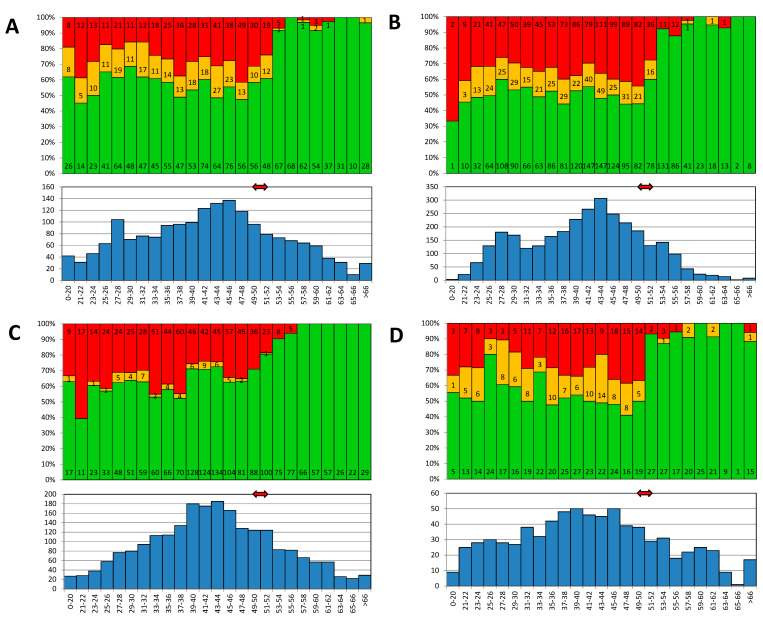
Distribution of measles antibodies positivity by age. Upper graphs: The proportion of positive (green), borderline (orange), and negative (red) results in individual age groups. Lower graphs: Histogram of the distribution of the population in individual age groups. Red arrow indicates the introduction of mandatory vaccination. (**A**) AGEL lab., (**B**) University Hospital Olomouc, (**C**) Mikrochem lab., (**D**) Šumperk Hospital.

The distribution of test results on a negative–borderline–positive scale and antibody levels in the individual age groups is shown in Figure 4. The levels of antibodies in all four laboratories were significantly different between age groups (*p* < 0.001). The significant association between the age and test result positivity was also confirmed by logistic regression (Table 2). Interestingly, in the case of University Hospital Olomouc lab, male sex was also significantly associated with test result positivity (OR = 1.2, 95% CI 1.02–1.47). 

A sharp decline in antibody levels is seen in people aged 52 years and younger, which corresponds to birth in 1967 and after. The portion of positive people in individual laboratories in this age group varied from 50.8% to 64.9%. The number of seropositives aged 53 years and over (born in 1966 and earlier) exceeded 92% in all laboratories; in two of them, the results were higher than 96% (Figure 3). This break correlates with the reduction in the natural circulation of the measles virus associated with the introduction of vaccination for children born in 1968.

**Figure 5:**
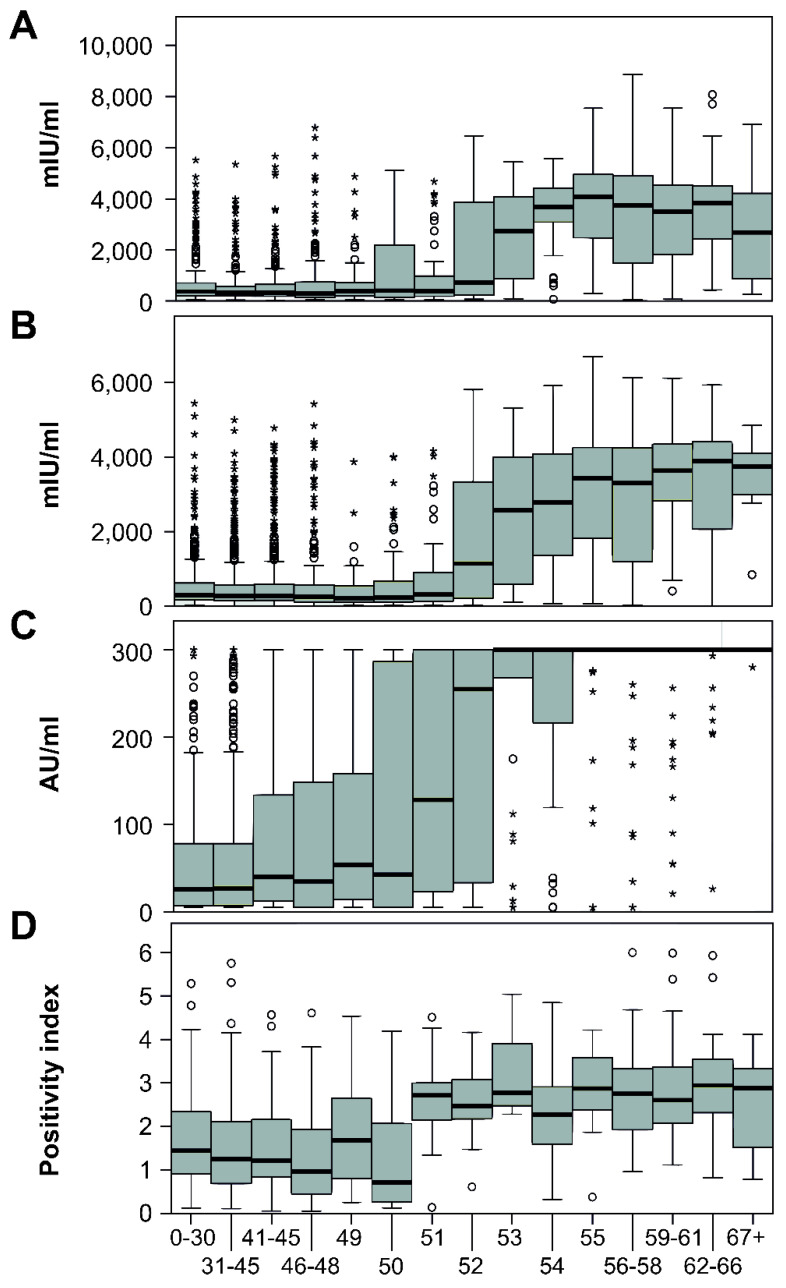
Distribution of test results in age groups. (**A**) AGEL lab., (**B**) Faculty Hospital, (**C**) Mikrochem lab., (**D**) Šumperk Hospital. The age groups in the charts are not uniform due to the emphasis on the trend after the age of 50. The units used differ between the first two and the third graph due to the difference in the methods used (ELISA with IU/mL vs. chemiluminescence with AU/mL). Symbols used: (○) outliers, (*) extremes.

In addition to a significantly higher percentage of seropositive individuals, an expressive increase in antibody concentrations was also noted (Figure 5). In laboratories using quantitatively evaluated EIA, there was an increase in the mean antibody concentrations in the people aged 53 and over, approximately five times—from 679.04 mIU/mL to 3306.15 mIU/mL in AGEL laboratories and from 545.86 mIU/mL to 2847.41 mIU/mL in University Hospital. In Šumperk Hospital, using EIA with relative evaluation, the average of the index was increased from 1.60 to 2.76. It was not possible to determine the average levels from the results of CLIA method used in Mikrochem laboratories due to the narrow dynamic range of the method. A total of 554 results were evaluated as more than 300 AU/mL and 356 as less than 5 AU/mL. However, a several-fold increase in the average value is noticeable on the graph here as well.

## 5. Discussion

According to the Global Vaccine Action Plan, measles was targeted for elimination in five WHO Regions by 2020 [20]. Unfortunately, this goal has not been achieved, and in recent years, the number of measles deaths has started to rise again. The number of deaths since 2016 has increased by almost 50% to 207,500 in 2019. Vaccination of the population with a single dose of measles vaccine has long stagnated at values around 85%, when values of up to 95% are required for reliable herd immunity due to calculation based on R_0_ number (who. int). Other risk factors include waning of immunity in some vaccinated individuals and gradually declining proportion of people with natural infection-induced high levels of antibodies. A new risk is the fact that, in 2020, vaccination was discontinued in 26 countries due to the COVID-19 pandemic. The Czech Republic is no exception in this unfavorable trend. According to the administrative control of the Ministry of Health, the vaccination coverage by MMR of the current year fell down from 98.02% in 2010 to 83.54% in 2017. The main reason is the loss of public confidence in the vaccination, associated with an overestimation of the side effects of vaccines and an underestimation of the severity of measles.

Given the current situation, the importance of surveying the state of immunity in the population is growing. Interest in testing the level of immunity is increasing both among individuals and among organizations such as medical facilities or rescue services. In the last two decades, several works dealing with the seroprevalence of measles antibodies have been published from data from the Czech Republic. A study from 2001 involving 3013 samples of individuals aged 1–64 years indicated a seropositivity of 78.4% and more in all age groups. Highest seropositivity rates were found in the population group aged over 35 years [21]. This result, when taking into account the time difference between the studies, corresponds to a group older than 53 years in our study. The second work evaluating 3111 samples from 2013 individuals in the same age range as in the previous study showed the average proportion of seropositivity 93%. Among persons older than one year, the positivity did not fall below 80.4%. In persons older than 45 years, in whom the authors assume natural exposure to the virus, a positivity of 97.7% was found. This age corresponds to the year of birth 1968 and earlier [22]. Measles IgG (II) EIA tests, Denka Seiken, Japan were used in both studies. According to the manufacturer’s instructions, samples with a result higher than 400 arbitrary units were considered positive, but no relation to the WHO standard is stated. Another recent work summarized the results from 1911 subjects aged over 18 years [23]. Overall, 83.3% of samples were evaluated as positive. In persons born before 1962, more than 96% of samples were positive. The EIA method with the diagnostic kit RIDASCREEN Measles Virus IgG (R-Biopharm, Darmstadt, Germany) was used. Results higher than 200 mIU/mL were reported positive, borderline 150–199 mIU/mL, and negative under 150 mIU/mL. In 2020, even three sero-surveys were published on the state of immunity against measles in hospital staff. In the first of them, seropositivity was noted in 54% of the participants [19], 74.6% was seropositive in the second one [24], and in the third, the published seropositivity rate was 93.7% [25]. 

We also found significantly different results between the results from the four laboratories analyzed in our study, although they all followed a similar population—mostly adults working in the healthcare, police, or fire departments in the same region. This difference may be partly due to differences in the age distribution of the populations, even if they are not too noticeable. Age distribution may largely explain the difference between the AGEL laboratories and the University Hospital Olomouc, which both used the same kit to detect antibodies. Although the difference in average age is only modest (42.15 vs. 40.88 years), the populations substantially differ in the proportion of subjects aged 53 years and more (20.08 vs. 11.28%). One-fifth of subjects tested in AGEL laboratories are thus people who probably underwent an infection and have consistently high levels of antibodies. However, the highest percentage of positivity was recorded in the Mikrochem laboratory, despite the fact that the proportion of people aged 53 and over is lower than in the AGEL (18.61%). The range of values evaluated as borderline can play a role in Mikrochem group, which is significantly narrower than in other laboratories. Some of the results, which would be evaluated as borderline by another method and therefore insufficient, with this method may already fall into a positive range.

As can be seen from our as well as previously published data, different laboratories using different detection kits provide distinct results on the overall positivity of antibodies in the study cohort. On the contrary, the same trend of distribution of antibody levels in age groups can be observed in all published reports. In persons born more than 2 years before the first mandatory vaccine introduction (1969), high levels of antibodies were found in more than 92% of persons in all studies. This corresponds to natural exposure to measles due to the circulation of the virus in the population. The differences in the overall percentage of positive results can be partly explained by the difference in the age distribution in each study cohort, where some studies followed only adults, some even children. However, the type of test set used and especially the method of evaluating the result will undoubtedly have a major influence. The current laboratory correlate of protection is based on a study that observed the effect of antibody levels on the risk of disease in blood donors in a measles outbreak at the University of Boston in 1985 [26]. All subjects with symptoms of the disease had a plaque-neutralizing assay detected antibody titer ≤120, but none of the subjects with a titer >120 became ill. This titer was extrapolated to 120 mIU/mL, and this value is currently generally accepted as the limit of protection against symptomatic disease. Concentrations less than 8 mIU/mL are considered to be completely seronegative [11,27,28,29]. 

Although standardization of immunoassay-based tests has advanced considerably and most manufacturers’ detection kits allow to obtain results in the International Units (mIU/mL) based on the WHO standard (currently the WHO 3rd international standard; The National Institute for Biological Standards and Control (NIBSC 97/648), different manufacturers still use different cut-offs to determine the positivity of the result, typically about 200–300 mIU/mL. A recently published comparison of different commercial immunoassays and plaque reduction neutralization test (PRN) found discrepancies between individual test kits, mainly in low-positive, equivocal, and high-negative ranges with false negativity in approximately 11% of samples. Good agreement was found for negative samples and for samples with intermediate to high antibody levels [29]. It can be assumed that, in some studies, the percentage of protected individuals is underestimated. From an epidemiological point of view, tendency to report uncertain values as negative is justified by the desire to eliminate possible false positive results. However, a false negative result may cause difficulties in revaccination effort with an attenuated MMR vaccine, as even low specific antibody levels may be sufficient to inactivate the vaccination virus leading to its failure. This phenomenon is well described in children with transplacentally transmitted antibodies, and it is a reason for vaccination only after reaching the first year of life [30,31]. Fiebelkorn et al. [32] studied the immunogenicity of the third dose of MMR vaccine in previously two-dose-vaccinated young adults. Neutralizing antibodies concentrations increased slightly but significantly compared with baseline after one month but declined almost back to baseline levels in one year. From subjects with a baseline antibody concentration ≤120 mIU/mL, only 67% had a concentration >120 mIU/mL one year after revaccination. Out of 617 study subjects, only 8 (1.3%) had ≥four-fold rises in measles antibody concentrations from baseline to 1 year post vaccination. One of them was the only person that was completely seronegative (<8 mIU/mL) before revaccination. Earlier study focused on the effect of different types of measles vaccines in monkeys passively immunized with serum from monkeys infected with measles virus. The serum level of measle antibodies in recipient animal sera as low as 100 mIU/mL abrogated effect of Schwarz strain attenuated vaccine and recombinant vaccinia virus expressing the F and H proteins of measles virus. However, the immunizing effect was maintained with the virus-free vaccine composed of purified H and F proteins [33]. These results suggest potentially only a limited booster effect of the currently used live vaccine in individuals with even low serum concentration of measles specific antibodies.

**Figure 6:**
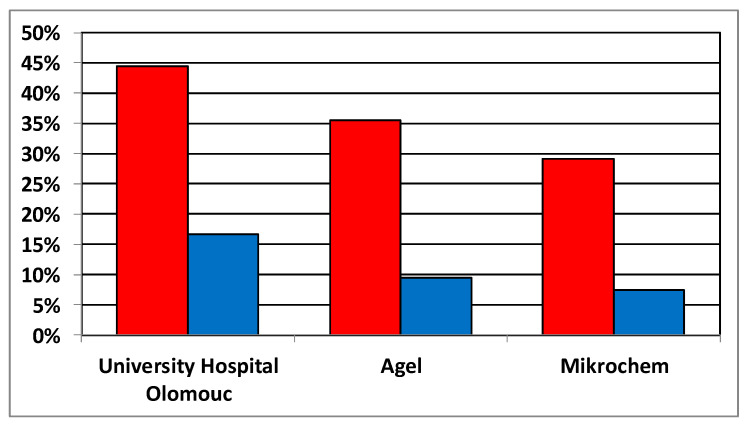
Comparison of the proportion of subjects indicated for revaccination based on the negative or borderline antibody levels issued based on manufacturer’s instructions in the preventive campaign (red) with the proportion of subjects having antibodies levels below generally accepted correlate of protection, i.e., <120 mIU/mL (blue). For the Mikrochem laboratories, the values were recalculated according to the ratio between the units used and the WHO standard provided in the manufacturer’s instructions. The data of the Šumperk Hospital laboratory could not be displayed due to the lack of connection to the mentioned standard.

Using data from three out of the four laboratories analyzed in our study, we compared the proportion of persons indicated for revaccination based on negative or borderline antibody levels issued in accordance with the manufacturer’s instructions with the proportion of persons who would be recommended revaccination based on the antibody level below the 120 mIU/mL limit (Figure 6). The comparison shows that the number of indicated persons decreases substantially from 44.5 to 16.7% at the University Hospital Olomouc, from 35.47 to 9.8% at AGEL laboratories, and from 29.2 to 7.41% at Mikrochem laboratories. This result suggests that the booster effect of live vaccine may be reduced by preexisting measles antibodies in a large proportion of revaccinated individuals. On this basis, we will try to evaluate the effectiveness of revaccination campaign in the University Hospital Olomouc in study aiming to assess the antibody levels after several years.

Commercially available immunoassays are currently virtually displacing methods based on the direct detection of viral neutralization effect in the routine determination of measles antibodies. However, the results of our as well as previous studies show their weakness in the determination of low levels of antibodies, which often occur in vaccinated individuals, caused in large part by inconsistencies in their interpretation. Our results confirm several times higher average levels of measles antibodies in people who have high probability of naturally experienced measles. In accordance with previously published results, the compared tests show a higher degree of agreement at higher antibody levels. The possible higher incidence of false-negative test results in vaccinated individuals may lead to an uneconomical allocation of resources to revaccination using live vaccine, which may fail in a significant proportion of individuals. In the future, it is highly desirable to pay increased attention to refining the correlation between the concentration of measles antibodies and protection against the disease. A great contribution to addressing the threat of future measles epidemics would be the development of a new generation of live virus-free vaccine able to induce booster effect even in individuals with low but still detectable levels of measles antibodies. One possibility is the introduction of vaccine-containing recombinant antigens, but following the experience gained with the vaccines against SARS-CoV-2, the development of innovative vaccines based on antigen coding nucleic acids sequences comes into play. 

## 6. Conclusions

In our work, we evaluated the results of seroprevalence studies from four laboratories from the Olomouc Region, Czech Republic, focused primarily on adults from selected professional groups. The studies were carried out on the basis of recommendations from local health authorities to identify critical infrastructure staff requiring measles revaccination. The results of 7962 people were processed, and three commercial methods were used in individual laboratories—CLIA and two different ELISA kits.

The main results of our work are as follows:

1. Despite the geographical and structural similarity of the tested groups of subjects, the portion of positive results in individual laboratories varied in a relatively wide range of 55.5% and 70.8%, depending on the test used. The differences between the laboratories were evaluated as statistically significant.
2. The results from all laboratories show the same trend, with a significantly higher proportion of positive results and higher levels of antibodies in people aged 53 and over who underwent measles before the introduction of measles vaccination. Post-vaccination antibody levels in the adult population are relatively stable over time.
3. Most available commercial diagnostic tests use higher levels of specific antibodies as the cut-off than the generally accepted correlate of measles disease protection (120 mIU/mL). Thus, the use of these tests in seroprevalence studies may underestimate the proportion of people immunologically protected from previous vaccination. This may lead to inefficient revaccination of part of the population with live vaccine, as even levels evaluated as negative result in the test may be sufficient to inactivation of the vaccine virus leading to revaccine failure.

## Figures and Tables

**Table 1:** Basic characteristics of the study population.

	AGEL Lab.	University H. Ol.	Mikrochem Lab.	Šumperk H.
N	1852	3093	2267	750
Average age (95% CI)	42.15 (41.61–42.69)	40.88 (40.55–41.21)	42.77 (41.7–42.6)	41.69 (40.84–42.54)
Median of age	43	42	43	41
Age range	0–78	20–72	1–91	19–82
Male gender	425 (22.94%)	622 (20.10%)	1293 (57.04%)	112 (14.93%)

**Table 2:** Significances of the test positivity and age groups.

Age Group	Odds Ratio	Sig.
0–20		0.000
20–30	1.280	0.317
30–40	1.080	0.305
40–45	1.117	0.116
45–48	0.940	0.481
48–49	1.028	0.855
49–50	1.075	0.625
50–51	1.395	0.040
51–52	2.448	0.000
52–53	7.412	0.000
53–54	14.120	0.000
54–55	17.371	0.000
55–56	9.103	0.000
56–57	41.099	0.000
57–60	25.549	0.000
60–62	47.578	0.000
62–65	75.081	0.000
65+	64.382	0.000

## Data Availability

Data available on request from the authors.
